# A survey of working conditions within biomedical research in the United Kingdom

**DOI:** 10.12688/f1000research.11029.2

**Published:** 2017-05-12

**Authors:** Nick Riddiford

**Affiliations:** 1 Institut Curie, Paris, 75248, France

**Keywords:** Biomedical science, working conditions, brain-drain, postdocalypse

## Abstract

**Background: **Many recent articles have presented a bleak view of career prospects in biomedical research in the US. Too many PhDs and postdocs are trained for too few research positions, creating a “holding-tank” of experienced senior postdocs who are unable to get a permanent position. Coupled with relatively low salaries and high levels of pressure to publish in top-tier academic journals, this has created a toxic environment that is perhaps responsible for a recently observed decline in biomedical postdocs in the US, the so-called “postdocalypse”.

**Methods:** In order to address the gulf of information relating to working habits and attitudes of UK-based biomedical researchers, a link to an online survey was included in an article published in the Guardian newspaper. Survey data were collected between 21 ^st^ March 2016 and 6 ^th^ November 2016 and analysed to examine discrete profiles for three major career stages: the PhD, the postdoc and the principal investigator.

**Results: **Overall, the data presented here echo trends observed in the US: The 520 UK-based biomedical researchers responding to the survey reported feeling disillusioned with academic research, due to the low chance of getting a permanent position and the long hours required at the bench. Also like the US, large numbers of researchers at each distinct career stage are considering leaving biomedical research altogether.

**Conclusions:** There are several systemic flaws in the academic scientific research machine – for example the continual overproduction of PhDs and the lack of stability in the early-mid stages of a research career - that are slowly being addressed in countries such as the US and Germany. These data suggest that similar flaws also exist in the UK, with a large proportion of respondents concerned about their future in research. To avoid lasting damage to the biomedical research agenda in the UK, addressing such concerns should be a major priority.

## Introduction

While there is no shortage of recent articles lamenting the current state of affairs in the scientific research machine ( Alberts *et al.*, 2014; Bourne, 2013; Gould, 2015; Powell, 2015; Sauermann & Roach, 2016), these have largely focussed on the US, and data relating to the UK are scarce. The general consensus from the US is that there is a growing workforce - particularly in the biomedical sciences - competing for a number of permanent research positions that has remained largely static since the 1980s ( Schillebeeckx *et al.*, 2013). Considering that the large majority of this workforce comprises PhD and postdoctoral researchers, who work almost exclusively on short-term, grant-funded contracts, competing for such positions often comes at the cost of stability, financial reward and any sense of work/life balance. Additionally, PhD programmes and postdoctoral posts tend to train scientists solely for a career in academic research, and neglect to equip them with a skill-set that would allow a smooth transition into gainful employment. Perhaps in response to these factors, after three decades of steady growth, the number of biomedical postdocs has started to decline in the US ( Garrison *et al.*, 2016). Such a “postdocalypse” is bad for the researchers squeezed out of a career in science, and bad for society as a whole.

Answering the call of several recent articles advocating for change within the system ( Benderly, 2015; Bourne, 2013; Gould, 2015; McDowell *et al.*, 2014; Powell, 2015), there have been a number of attempts to quantify factors contributing to such a trend ( McDowell & Heggeness, 2017; Powell, 2016; Sauermann & Roach, 2016). However, while such data are highly revealing, there is a general lack of UK-centric data, and almost a complete absence of the strong advocacy groups for young scientists that have been so successful elsewhere ( Cain *et al.*, 2014; McDowell *et al.*, 2014). Consequently, this article attempts to plug this gap, and provide a data point for UK-based biomedical scientists. Here, I present an in-depth analysis of survey data collected in response to a recent article calling for change within the UK biomedical system ( Riddiford, 2016a). The survey was answered by 1,128 scientists as of 6 ^th^ November 2016, and suggests that trends observed in the US are broadly echoed in the UK. While such data are subject to several interpretational caveats associated with survey results – most importantly that respondents are necessarily biased towards those who have read the survey – they are important in illustrating broad themes, and highlighting areas of interest for any future work. 

## Methods

### Survey design

A ten-question survey was designed to formally evaluate the working habits of biomedical researches. A link to the survey was included in a news article published in the Higher Education section of the Guardian newspaper on 21 ^st^ March 2016 ( Riddiford, 2016a). While the primary intention was to gather information relating to UK-based biomedical scientists, the survey was also open to non-UK-based scientists from a broad range of backgrounds for comparison. The first three questions “what position are you?”, “broadly, what discipline do you work in?” and “what country do you work in?” aimed to serve as a filter to ensure the accurate analysis of UK-based biomedical scientists at different stages of their career. The following three questions “how many countries have you worked in over the past five years?”, “how old are you?” and “how long have you held this level of position?” aimed to construct a demographic census of the respondents, and to enable comparison between specific age groups. The next three questions focussed on the conditions scientists work under, asking “how many hours did you work last week?”, “how many days did you work last week?”, “what’s your annual salary in pounds sterling?”. The final question “how comfortable do you feel about your long-term prospects in research?” gave respondents the opportunity to select multiple responses, and those selecting the answer “not at all – I’m planning on leaving research” were invited to expand on their answer, and detail any factors contributing to this decision. The full list of questions and accompanying answer options are available in Supplementary File 1. The survey is still active and is hosted by Survey Monkey ( https://www.surveymonkey.co.uk/r/HBP6NXX).

### Data analysis

**Figure 1.**
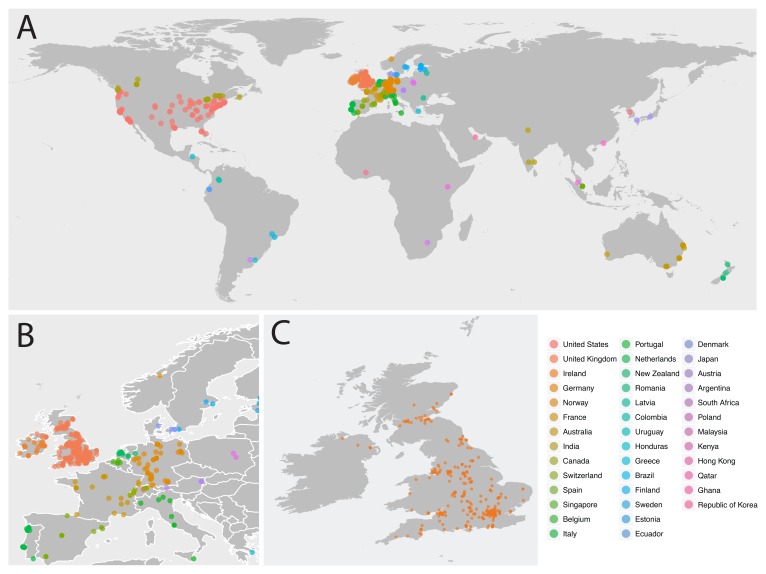
Distribution of responses based on geolocation of IP address. Each point shows a single IP address and points are coloured by country. **A** shows IP addresses on a global map, whereas **B** and **C** show distributions across Western Europe ( **B**) and the United Kingdom ( **C**). Country boarders are shown as white lines in **A** and **B**.

To capture as many responses as possible, data were collected between 21 ^st^ March 2016 and 6 ^th^ November 2016 ( Dataset 1; Riddiford, 2017). In this time period, the survey was answered by 1,128 scientists. Initially, to give an overview of the distribution of respondents by country, IP addresses associated with each respondent were used to query a free-to-use geolocation database ( http://freegeoip.net). Resulting data were then plotted on a world map, and focussed on Europe and the UK for a finer distribution of IP addresses. All the code used to generate the plots shown in Figure 1 is deposited in the Github repository https://github.com/nriddiford/Mapped-IP-addresses.

Next, data were filtered to select only for responses from UK-based biomedical researchers (Q2 response: “biomedical sciences”; Q3: “UK”) to give a broad overview of working conditions within this cohort. Data were then further filtered to provide a career-stage-specific profile for each of the major tiers of an academic research career; the PhD, the postdoc and the principal investigator (Q1: “PhD”, “postdoc” and “principal investigator, permanent contract” or “principal investigator, non-permanent contract”). Data for each discrete profile were then analysed using a custom Perl script ( Supplementary File 2) to parse downloaded data and include non-standard question answers (i.e. where respondents opted to specify a non-listed answer, or to elaborate on their selected response) in the analysis. Average salaries for each group were calculated by using the mean number of hours and salary for each cohort as queries for the web-based UK salary calculator ( http://www.thesalarycalculator.co.uk/salary.php). The average salary for PhD students was calculated using the option “no NI”.

For the 299 respondents who provided a written answer to describe in detail the reasons they were planning on leaving research (Q10: “not at all – I’m planning on leaving research”), four statements were selected for each career stage as being broadly representative of the issues addressed by others in the same cohort, and are presented in Box 1– Box 3. The complete unanalysed data set for responses collected within the stated time period can be found in Dataset 1 ( Riddiford, 2017; answers compromising the anonymity of respondents [IP address and personal comments] are not included).

Box 1. **Selected representative statements given in the survey by PhD students planning on leaving academia**

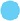
   “I am told to be ambitious yet there just aren't enough jobs for us all to be ambitious. Too much is down to chance.”

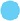
   “The system is broken and yet is perpetuated as it is the lucky (and clever) few who make it to the top and tell everyone it will work out if you work hard. The simple fact is: For most people it will not.”

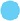
   “The career prospects, a decade of uncertain employment and relatively low pay mean getting out early is a priority for me and many others from my department.”

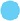
   “It's essentially a pyramid scheme and once you realise the stats, you start looking for safer alternatives.”

Box 2. **Selected representative statements given in the survey by postdocs planning on leaving academia**

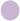
   “I'm unwilling to compete against people who will work 12+ hours a day, 7 days a week. The structure of scientific research makes a future in academia look incredibly unappealing.”

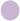
   “I've realised it's a pyramid scheme and I'm never going to get a lectureship so I've decided to leave for more stability. Also, I just can't move again, eventually I want to stay in one place for more than 3 years!”

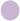
   “I am not prepared to uproot my family again for another temporary post, so I am not willing to relocate.”

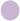
   “I think pursuing a permanent position in academia is effectively gambling with my future.”

Box 3. **Selected representative statements given in the survey by PIs planning on leaving academia**

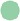
   “Teaching standards are plummeting, and research funding is nearly impossible to gain. University education and research is about to collapse. It is not a viable career in the UK, despite our dominance in research."

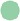
   “If I cannot secure funding in the next two years, I will face losing the job and leaving research."

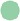
   “When I finally became a PI, I realised that the view I had of academic life was very naive. I can only do research that can be funded. There is not a single day that I do not worry about the project, competition, funding, publications etc."

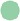
   “I have such a heavy teaching load I can't do research as well."

## Results

### A global view of survey respondents

A total of 1,128 responses were collected between 21 ^st^ March 2016 and 6 ^th^ November 2016, including responses from 40 countries ( Figure 1 A), although the large majority were distributed across Western European countries and North America ( Figure 1 A, B). Of the total responses, 667 were based in the UK ( Figure 1 C).

### A general profile of biomedical researchers

Of the 1,128 respondents, 900 classified themselves as biomedical scientists, 37% of whom reported having worked more than 50 hours in the week preceding the survey (12%, ≥ 60 hours). Perhaps more striking was the finding that 53% reported working more than five days the week before they answered the survey and that 15% worked every day that week ( Supplementary File 1). Only 16% reported receiving an annual salary in excess of £35,000. Almost all of the respondents were PhD students or postdocs, and 98% were employed on short-term contracts.

### Discrete profiles for UK-based biomedical researchers

The data were then filtered to select only for UK-based biomedical researchers, yielding a total of 520 responses, comprising 306 PhD students, 142 postdocs, 30 PIs and 42 working in roles outside of these three categories (such as research assistant), that were not included in further analyses.

**Figure 2.**
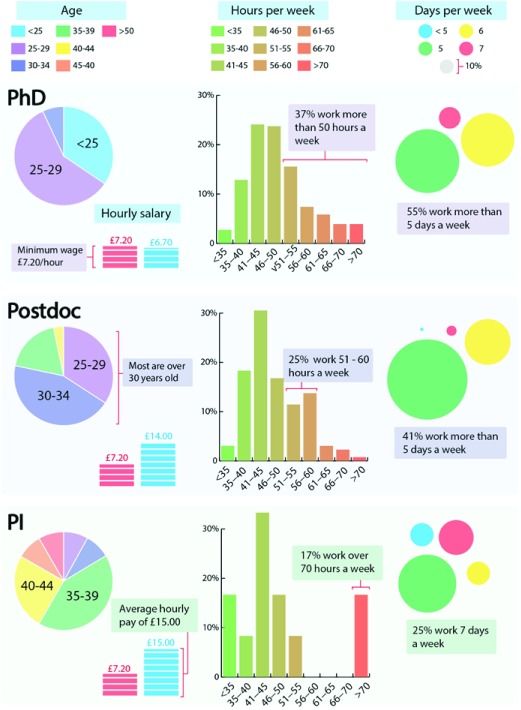
Graphical representation of analysed survey data. The data are presented for three discrete career stages, the PhD (as shown in light blue box ‘ **PhD**’), the postdoc (light purple box ‘ **postdoc**’) and the PI (light green box ‘ **PI**’). Venn diagrams show the distribution of respondents per cohort across age categories, with each colour representing a discrete age range (light blue: younger than 25; purple: 25–29; dark blue: 30–34; green: 35–39; yellow: 40–44; orange: 45–50; red: older than 50). Hourly salaries are shown as stylised bar charts, where the height of the bar represents hourly salary. In each case, the average salary is shown in light blue, contrasted with the minimum hourly salary shown in red. The average number of hours worked per week for each group is represented as a bar chart. In each case, the percentage of respondents per category is shown plotted against the number of hours worked per week. The number of days worked per week are represented as bubbles for each group, where bubbles are coloured by category (blue: fewer than 5 days per week; green: 5 days per week; yellow: 6 days per week; red: 7 days per week). The size of each bubble represents the relative number of respondents per group in each category. The legend shows a reference bubble (coloured grey) representing a value of 10 percent.

***PhD students.*** The majority of respondents to the survey were PhD students (59%), representing the youngest, and most mobile cohort, with 94% aged between 25–29 and 35% having worked in two or more countries over the past five years ( Dataset 1 ( Riddiford, 2017); Supplementary File 1). On average, they also reported working more hours per week than other cohorts (37% work over 50 hours a week) and the majority worked more than five days in the week before answering the survey (55%, > five days; 16%, seven days; Figure 2 - ‘PhD’). UK-based PhD students are typically funded via a tax-free stipend of between £13,000 and £20,000, which equates to an hourly salary of £6.70 (average working week reported: 48 hours; average salary: £17,000 [tax-free; excluding NI contribution]). PhD students are funded on a short-term basis, and 92% of PhD respondents have been at their current level of position for fewer than four years.

In response to the question “How comfortable do you feel about your long-term prospects in research?” 5% answered “comfortable”, with the vast majority expressing major concerns about one or more work-related factors. The most common reason for respondents’ lack of comfort in the prospect of a career in research was “it’s too competitive, and there aren’t enough jobs” (63%), followed by “I don’t make enough money” (45%). Surprisingly, only 28% plan on leaving academia (see Box 1 for several respondent-provided statements).

***Postdoctoral researchers.*** The next rung on the academic ladder - and therefore the next discrete cohort analysed - is the postdoctoral research fellowship (“postdoc”), and accordingly this cohort generally comprised older respondents (65% age 30 or older; Figure 2 - ‘Postdoc’). Like PhD students, roughly a third reported having worked in two or more countries over the past five years (33%). While postdocs are also employed on a short-term basis, the number of respondents who reported being employed at the same level for four or more years was drastically higher than for PhD students (≥ four years: postdoc, 32%; PhD, 11%; ≥ ten years: postdoc, 4.5%; PhD, 0.3%), almost certainly reflecting the growing necessity of pursuing multiple postdocs on the path to becoming a full faculty member ( Bourne, 2013).

Also like PhD students, postdocs work long hours - 79% reported working more than 40 hours a week, and 41% for more than five days a week. Despite their age, experience and work ethic, the average salary for biomedical postdocs in the UK is relatively low, with 75% of postdocs earning between £26,000 and £35,000 (4.5% earn more than £41,000), which constitutes an average hourly salary of approximately £14.00 (average working week reported: 45 hours; average salary: £33,000). However, despite only 7% describing themselves as “comfortable” in their long-term prospects for a career in research, only 30% plan on leaving academic research (see Box 2 for several representative reasons). The large majority that didn’t feel comfortable in a future in research and felt that they were competing for too few jobs (66% answered “It’s too competitive, and there aren’t enough jobs”) and working too hard (33% answered “I can’t keep working this hard”).

***Principal investigators.*** The final group comprises those who identified as being a principal investigator (“PI”), and therefore represent an older and more stable cohort that PhD students or postdocs. In total, 63% of respondents in this group were employed on a permanent contract, and only 20% reported working in more than two countries over the last five years. In addition, 80% were over 35 years old and 48% reported being employed at the same level for four years or more (≥ ten years: 28%; Dataset 1 ( Riddiford, 2017)). However, this category was vastly underrepresented in the survey data – only 30 individuals responded in total, and only eight were aged over 45 years – representing a major caveat in the interpretation of such data. While such low numbers are insufficient to draw any major conclusions, the data collected do provide some insight into the working habits of UK-based biomedical PIs, and particularly of younger individuals (52% employed at this level for ≤ four years). In particular, 17% in this cohort reported working over 70 hours in the week preceding the survey, and 25% worked a seven-day week ( Figure 2 - ‘PI’).

Like PhD students and postdocs, the average salary from this group was relatively low (£41,000), which is particularly striking when considering the level of experience required to reach such a position, which equates to an hourly salary of just £15 (average working week reported: 49 hours; average salary: £41,000). Accordingly, a low salary was cited as a cause for concern by 38% of respondents (Q10: “I don’t make enough money”), while more respondents felt that their work/life balance was unsustainable (46%; “I can’t keep working this hard”). As in the earlier stages of a research career, roughly a third (31%) plan on leaving research for reasons such as those given in Box 3.

Raw data from the survey (anonymity-compromising information has been removed, see Methods). Click here for additional data file. Copyright: © 2017 Riddiford N 2017 Data associated with the article are available under the terms of the Creative Commons Zero "No rights reserved" data waiver (CC0 1.0 Public domain dedication).

## Discussion

The survey data presented here provide a rare and valuable insight into the working conditions of UK-based biomedical researchers. While there has been a recent surge in data collection focussing on the scientific research community - and largely in the biomedical sector ( McDowell & Heggeness, 2017; Powell, 2016; Sauermann & Roach, 2016) - these tend to be concentrated on the US workforce, and data pertaining specifically to the UK are scarce. Therefore, the data presented here are intended to fill this void, and provide a foundation for future discussion relating to biomedical researchers in the UK. While the survey was largely intended to collect responses from UK-based researchers, it was also answered by individuals across 40 countries ( Figure 1A), indicating that concern for such issues is not confined to the US and Europe.

Overall, the data presented here suggest a large faction of biomedical researchers working in the UK are deeply concerned about their long-term future in research. In each discrete career stage analysed, roughly equal numbers (PhD: 28%; postdoc: 30%; PI: 31%; Dataset 1; ( Riddiford, 2017)) plan on leaving academic research, largely due to the lack of job opportunities, and the degree of competition involved in attaining a permanent position. Such findings are largely consistent with the number of scientists reported to be planning on leaving research in the US ( Sauermann & Roach, 2016), and represent a major problem - the “brain-drain” - facing biomedical research ( Benderly, 2015; Healy, 1988).

The data also suggest that biomedical scientists in the UK are working long hours and over weekends for relatively little financial reward: 53% worked more than five days in the week before they took the survey, and only 16% reported receiving an annual salary of over £35,000. A recent online poll of readers conducted by the journal *Nature* revealed that almost 40% of the 12,000 respondents worked more than 60 hours a week on average ( Powell, 2016), a substantially higher number than that found in this survey (12% across all career stages). One explanation is that while the *Nature* poll asked readers (from all scientific disciplines) to report their average working week, the survey presented here instead asked respondents to report the number of hours worked in the week immediately preceding the survey, and to estimate an average only if this value was atypical. This approach was adopted to limit over-estimation and to provide a more accurate dataset. The same *Nature* poll also reported that almost two thirds of readers have considered leaving research altogether, and that 15% have actually left, again, far higher than numbers reported here ( Powell, 2016). While approximately 30% of UK-based biomedical scientists surveyed here reported their plans to leave research, it is possible that this figure is somewhat inflated. Firstly, as with any survey or poll, individuals who do not engage are just as illustrative as those who do. It is likely that there exists a population of biomedical researchers who are satisfied enough with their work/life balance that that they choose not to engage with articles addressing such issues, which would tend to dilute more positive views. Secondly, despite approximately 30% of respondents surveyed here stating their intention to leave research, it is probable that some fraction of these will decide to remain, and the number who actually do leave may well be lower. However, considering that the majority of respondents were PhD students, it is likely that many will indeed pursue a career outside of academia – a Royal Society report from 2010 estimates that 53% of science PhD students do not pursue an academic career – making a leaving rate of 30% seem more likely ( The Royal Society, 2010).

Nonetheless, the almost 300 personal testimonials describing why researchers were planning on leaving are striking. Almost all of these reiterated the same concerns: that continuing in research was not only gambling with their future, but that it was also a bad bet to make in the first place. Many also noted that the hypercompetition ( Alberts *et al.*, 2014) involved in attaining a faculty position diluted their bargaining power, and drove up the need to sacrifice any sense of work/life balance. For many, this sacrifice is just not a viable option, and rather than facing the prospect of effectively being forced out of a career in scientific research, often at late stages of their careers ( Riddiford, 2016b), they are exiting on their own terms.

Given the febrile political landscape in the UK and elsewhere, it is perhaps more crucial than ever that the biomedical research community in the UK rally together to ensure that pursuing a career in biomedical research does not require one to gamble with one’s future career prospects. In addition, those who make this bet should do so in full knowledge of the employment landscape within academic research. 

## Ethics statement

Considering the absence of identifying information in data published here, and the non-sensitive nature of the survey, no ethical approval was sought for this study. No information presented here can be used to identify survey participants, and in accordance with SurveyMonkey’s data privacy policy ( https://www.surveymonkey.com/mp/policy/privacy-policy/), is not accessible to third parties.

## Data availability

The data referenced by this article are under copyright with the following copyright statement: Copyright: © 2017 Riddiford N

Data associated with the article are available under the terms of the Creative Commons Zero "No rights reserved" data waiver (CC0 1.0 Public domain dedication). 

Dataset 1: Raw data from the survey (anonymity-compromising information has been removed, see Methods). doi, 10.5256/f1000research.11029.d153379 ( Riddiford, 2017).

## Supplementary material

Supplementary File 1: The complete survey.

Supplementary File 2: Perl scripts used to analyse days and hours worked.
