# Supplementary material for: A survey of working conditions within biomedical research in the United Kingdom
Source: F1000Res. 2017 May 12;6:229. Originally published 2017 Mar 7. [Version 2] doi: 10.12688/f1000research.11029.2 (PMC5627577; doi:10.12688/f1000research.11029.2)
Supplement: Supplementary file 2 [file f1000research-6-12484-s0001.tgz › 96391988-d1ff-4314-85b5-60efa5834690.docx]

| **A survey of scientist satisfaction** |
| --- |

**1. What position are you?**

1. PhD researcher
2. Post-PhD, unemployed
3. Postdoc
4. Postdoc, between contracts
5. Lecturer, short-term contract
6. Principal Investigator, non-permanent contract
7. Principal Investigator, permanent contract
8. I have left academia, but I am filling out this survey according to my most recent post (please indicate)
9. If you don’t fit into these categories, please expand below

**2. Broadly, what discipline do you work in?**

1. Biomedical sciences (cell biology, developmental biology, molecular biology, bioinformatics etc)
2. Biology (ecology, evolution etc)
3. Physics
4. Chemistry
5. Maths
6. Other (please specify)

**3. What country do you work in?**

1. UK
2. Europe (non-UK)
3. US
4. Canada
5. Other (please specify)

**4. How many countries have you worked in over the past 5 years?**

1. One
2. Two
3. Three
4. Four
5. Five or more

**5. How old are you?**

1. Under 25
2. 25 – 29
3. 30 – 34
4. 35 – 39
5. 40 – 44
6. 45 – 49
7. 50 or older

**6. How long have you held this level of position?**

1. Under a year
2. 1 - 3 years
3. 4 - 6 years
4. 7 - 9 years
5. 10 years or more

**7. How many hours did you work last week?**

1. Fewer than 35
2. 35 - 40
3. 41 - 45
4. 46 - 50
5. 51 - 55
6. 56 - 60
7. 61 - 65
8. 66 - 70
9. More than 70
10. Last week was not a normal week. I usually work [specify] hours per week

**8. How many days did you work last week?**

1. Fewer than 5
2. 5
3. 6
4. 7
5. Last week was not a normal week. I usually work [specify] days per week

**9. What’s your annual salary in pounds sterling?**

1. I’m currently not paid
2. PhD stipend 13,000 - 18,000 (tax free)
3. PhD stipend 18,000 - 20,000 (tax free)
4. 21,000 - 25,000
5. 26,000 - 30,000
6. 31,000 - 35,000
7. 36,000 - 40,000
8. 41,000 - 45,000
9. 46,000 - 50,000
10. 51,000 - 60,000
11. 61,000 - 70,000
12. 71,000 - 80,000
13. 81,000 - 100,000
14. 101,000 or more
15. Other (please specify)

**10. How comfortable do you feel about your long-term prospects in research? (Select all that apply)**

1. Comfortable
2. Not very comfortable - I can’t keep working this hard
3. Not very comfortable - I don’t make enough money
4. Not very comfortable - I can’t, or won’t, relocate
5. Not very comfortable - It’s too competitive, and there aren’t enough jobs
6. Not at all - I’m planning on leaving research (please expand below)
