# Supplementary material for: A survey of working conditions within biomedical research in the United Kingdom
Source: F1000Res. 2017 May 12;6:229. Originally published 2017 Mar 7. [Version 2] doi: 10.12688/f1000research.11029.2 (PMC5627577; doi:10.12688/f1000research.11029.2)
Supplement: Peer review history

# Peer review: Referee response for version 2

**DOI:** 10.5256/f1000research.12484.r22658

**Published:** 2017-05-22

**Authors:** Jessica K. Polka

**Affiliations:** 1 Whitehead Institute, Cambridge, MA, USA

The manuscript has been improved throughout (including a beautiful new figure), but several of my concerns have not yet been addressed. 

 For example, please make it explicit that your survey is intended to cover experiences within the UK *academic* biomedical system.

 "Researchers" is misspelled in the first sentence of "survey design," and the sentence beginning with "the large majority that" has a grammatical error. In this sentence, it would be helpful to remind the reader that question 10 allowed respondents to select all that apply.

 Figure 2 is still difficult to read - please see my original comments.

I have read this submission. I believe that I have an appropriate level of expertise to confirm that it is of an acceptable scientific standard, however I have significant reservations, as outlined above.

# Peer review: Referee response for version 1

**DOI:** 10.5256/f1000research.11897.r20903

**Published:** 2017-03-28

**Authors:** Kearney T. W. Gunsalus

**Affiliations:** 1 Department of Biology, Siena College, Loudonville, NY, USA

This paper addresses an important gap in the available data regarding the biomedical workforce in the UK. Although larger studies are needed (both in the scope of the questions asked and with a larger and more representative population of respondents), this survey is a nice example of how members of the community can begin to address the gaps in existing data collection and dissemination efforts.  

Abstract

 Please include the number of respondents analyzed and mention something about how the survey was advertised/the target audience. (The caveats about survey responses necessarily being biased to those who were aware of it and cared enough to take the time to respond are buried fairly far into the discussion, and it would be helpful to make some reference to this a little earlier in the paper.)

Data presentation

 Please state clearly throughout the text the number of responses analyzed in each category. (How many UK-based biomedical researchers responded to the survey? How many PhD student and postdoctoral respondents were there? Etc.)

 It would be interesting to include a figure showing responses to the final question (how comfortable do you feel about your long-term prospects in research?"); as respondents had the option to select multiple answers, it would be nice to see the percentage selecting each of the possible answers.  

 For clarity, the colorblind, and those who still prefer to print papers in black and white, it would be preferable to directly label the data in figure 1, rather than using the key. Please label the percentage of respondents in each age group; and label graph axes. Though more typical for an infographic than a figure in a paper, I do like the text boxes highlighting the take-home message for each panel.  

 I also initially found the visual representation the hourly salary data somewhat confusing; it might make more sense to make the "hourly salary" its own panel, with bars for minimum wage, PhD, postdoc, and PI average hourly pay (rather than showing the minimum wage three times). The hourly salary numbers could be included in or above each bar.  

 Could you clarify the way average hourly wages are calculated? I noticed for grad students, you assumed a 48-hour work week, while for postdocs the assumption was a 45-hour work week, and I didn't see a number for PIs. Were the assumptions supposed to represent a "typical" respondent, or an average?  

Future directions for the survey

 I hope that data collection and analysis for this project will continue. I have a few minor suggestions, should the survey be revised.  

- I found it somewhat confusing that the category for (predoctoral) graduate students appears to be "PhD researcher."
- While I understand the importance of keeping such a survey short, it might be helpful to collect some additional demographic data, such as gender, relationship/marital status (and if partnered, the partner's salary and discipline), and number of children.  

Overall, this work addresses an important knowledge gap. I hope data collection will continue and that in future the survey questions can be expanded and the survey itself advertised more broadly.

I have read this submission. I believe that I have an appropriate level of expertise to confirm that it is of an acceptable scientific standard, however I have significant reservations, as outlined above.

# Peer review: Referee response for version 1

**DOI:** 10.5256/f1000research.11897.r20768

**Published:** 2017-03-20

**Authors:** Jessica K. Polka

**Affiliations:** 1 Whitehead Institute, Cambridge, MA, USA

This survey represents an important contribution to our understanding of career satisfaction among early career researchers. As you note, many efforts have focused on the US, so this study is especially valuable in light of its focus on the UK. However, the opt-in nature of the survey should be disclosed in the abstract, and several other parts of the manuscript could be productively modified.

**Methods**

- Please explain how the survey was advertised and what target audiences were likely reached.
- Since you have IP addresses, can you report how many of the responses came from within the UK and how many were from academic institutions? The former would be essential support for the claim that the report is representative of ECRs in the UK.
- Furthermore, can you compare age and other factors to any known statistics to evaluate how representative your sample is in these dimensions?

**Results**

- Please clarify whether the term “research” is used to mean academic research or research in industry as well. If the latter, did any of the survey respondents identify as industry researchers? For example, at the end of the section on PhD students, you write that 28% plan on leaving academia, yet the question asks about research - a very important distinction. Furthermore, I’m not sure that the fact that “only 28%” are planning on leaving is surprising, since the respondents did not provide information about their available alternatives.
- In the postdoc section, the statement “the large majority that didn’t feel comfortable in a future in research felt that they were working too hard” does not make sense at only a 33% response rate.
- Throughout, it would be helpful to provide the actual # of responses received, especially when discussing a fraction of a category (for example, X% of postdocs, etc).

**Discussion**

- Science can offer non-financial rewards, such as the pleasure of doing research and a relatively high level of respect. Therefore I suggest providing a caveat {indicated} to the sentence: “Working long hours and over weekends for relatively little {financial} reward”
- Regarding the statement “it is probable that some fraction of [researchers stating their intention to leave] will decide to remain, and the number who actually do leave may well be lower.” Rather than speculate, can you compare this to existing data on attrition rate, for example figure 1.6 from the 2010 Royal Society report “The Scientific Century” ^1^?

**Figures**

- Box 1-3: The colored bullet points are distracting - does the color code have meaning?
- Figure 1: This graphic is extremely difficult to read. Please label the pie chart sections directly (or better yet, make it a histogram) and provide axis titles and labels for all of the graphs. This will make the legends unnecessary. The “days per week” visualization would be much better represented by a distribution. PhD (which is ambiguous and should perhaps be PhD student), Postdoc, and PI labels are unnecessarily large.
- The data on comfort with long term prospects in research are very interesting. I would like to see a graphical representation of this as well.

I have read this submission. I believe that I have an appropriate level of expertise to confirm that it is of an acceptable scientific standard, however I have significant reservations, as outlined above.

## References

[1] The Scientific Century: securing our future prosperity. *The Royal Society*.2010;
